# Supplementary material for: A Likelihood Approach for Real-Time Calibration of Stochastic Compartmental Epidemic Models
Source: PLoS Comput Biol. 2017 Jan 17;13(1):e1005257. doi: 10.1371/journal.pcbi.1005257 (PMC5240920; doi:10.1371/journal.pcbi.1005257)
Supplement: S1 File — (TAR.GZ) [file pcbi.1005257.s014.tar.gz › HSPH_Online-SI-Revision/EnKF07/n100-severe/n100-severe_EnKF2_table0.pdf]

1	newly 3 weeks spec	5129.	1488.59	{96., 1501.}	99.0893	1.16438	newly 3 weeks cumul	17951.	4563.03	{534., 5073.}	93.0349	1.04999	newly 5 weeks spec	3152.	797.2	{9., 2895.}	83.5863	1.25496	newly 5 weeks cumul	25181.	6396.42	{894., 8201.}	91.6201	1.03316	newly 50 weeks cumul 33334. 19086.1 {1341., 85171.} 96.5994 0.897856
2	newly 3 weeks spec	3271.	1430.33	{122., 1413.}	99.7832	0.907623	newly 3 weeks cumul	16291.	6903.45	{728., 6422.}	98.3979	0.903217	newly 5 weeks spec	952.	648.39	{52., 769.}	105.259	0.778908	newly 5 weeks cumul	19055.	8493.17	{944., 8290.}	98.766	0.877816	newly 57 weeks cumul 20150. 10046.2 {1030., 10081.} 98.1328 0.813246
3	newly 3 weeks spec	3370.	1915.67	{250., 2453.}	86.4021	0.693201	newly 3 weeks cumul	19722.	11679.	{2893., 14312.}	84.797	0.548447	newly 5 weeks spec	834.	610.37	{13., 1259.}	71.8573	0.778253	newly 5 weeks cumul	22331.	13332.4	{3276., 14481.}	82.3662	0.527133	newly 58 weeks cumul 23238. 14578.2 {3394., 17667.} 78.9999 0.490398
4	newly 3 weeks spec	5397.	2274.2	{65., 11078.}	101.191	1.16399	newly 3 weeks cumul	21357.	13443.1	{493., 40221.}	98.1699	0.841316	newly 5 weeks spec	2735.	604.82	{7., 818.}	95.9949	1.60664	newly 5 weeks cumul	27895.	15178.9	{603., 59039.}	97.1626	0.90481	newly 56 weeks cumul 32739. 16137.1 {603., 60007.} 96.0415 0.941664
5	newly 3 weeks spec	4803.	2753.63	{168., 13649.}	102.259	0.925705	newly 3 weeks cumul	16297.	6214.16	{815., 20313.}	80.8948	0.841207	newly 5 weeks spec	3233.	1904.29	{92., 7780.}	95.7473	0.944302	newly 5 weeks cumul	23516.	10809.3	{1158., 51133.}	87.8548	0.852119	newly 50 weeks cumul 34577. 22212.3 {1437., 79910.} 90.4545 0.827793
6	newly 3 weeks spec	6383.	2545.49	{87., 10610.}	95.877	1.08857	newly 3 weeks cumul	23683.	16997.6	{1230., 85181.}	103.981	0.756272	newly 5 weeks spec	2924.	606.42	{9., 1226.}	94.0506	1.57678	newly 5 weeks cumul	31005.	18783.9	{1319., 97584.}	99.8073	0.81119	newly 56 weeks cumul 36697. 20098.5 {1331., 98445.} 96.7983 0.84465
7	newly 3 weeks spec	5854.	1293.71	{237., 1680.}	88.8861	1.01617	newly 3 weeks cumul	26186.	7217.97	{1176., 8008.}	93.3173	0.986176	newly 5 weeks spec	2014.	487.52	{74., 941.}	82.0248	0.927473	newly 5 weeks cumul	31770.	8431.75	{1474., 9475.}	90.6923	0.958086	newly 57 weeks cumul 34576. 10328.4 {1905., 11919.} 88.459 0.887002
8	newly 3 weeks spec	4856.	875.99	{94., 1721.}	95.2477	1.27737	newly 3 weeks cumul	17949.	6535.83	{744., 7957.}	93.5209	0.919155	newly 5 weeks spec	2677.	290.16	{6., 231.}	99.4905	1.79821	newly 5 weeks cumul	24358.	7318.12	{825., 10035.}	93.8855	0.998816	newly 53 weeks cumul 30079. 7739.81 {846., 11893.} 93.9632 1.06202
9	newly 3 weeks spec	4469.	1359.9	{254., 2362.}	77.7583	0.807772	newly 3 weeks cumul	27073.	9509.51	{3206., 21595.}	72.833	0.616618	newly 5 weeks spec	876.	513.29	{6., 1168.}	68.8436	0.861572	newly 5 weeks cumul	30073.	10818.2	{3486., 25955.}	71.4919	0.594024	newly 58 weeks cumul 30570. 12043.4 {3519., 26477.} 69.6818 0.555855
10	newly 3 weeks spec	5097.	855.88	{22., 670.}	103.398	1.63028	newly 3 weeks cumul	20668.	2428.98	{201., 2274.}	95.3244	1.44945	newly 5 weeks spec	2025.	273.58	{2., 520.}	96.4405	1.69913	newly 5 weeks cumul	25993.	3244.03	{262., 3547.}	96.1957	1.46001	newly 51 weeks cumul 29196. 3994.96 {284., 7640.} 94.0895 1.40851
11	newly 3 weeks spec	4623.	1275.36	{239., 1630.}	89.3281	0.930489	newly 3 weeks cumul	21770.	6901.57	{1002., 7445.}	94.5592	0.927013	newly 5 weeks spec	1559.	468.03	{63., 919.}	79.0847	0.828638	newly 5 weeks cumul	26128.	8076.92	{1361., 8723.}	91.4792	0.897205	newly 57 weeks cumul 28027. 9923.66 {1932., 11569.} 89.6656 0.828486
12	newly 3 weeks spec	5357.	688.42	{118., 1171.}	92.6239	1.25503	newly 3 weeks cumul	18503.	2344.78	{430., 2640.}	89.8485	1.17003	newly 5 weeks spec	3340.	578.39	{17., 1083.}	90.7332	1.30927	newly 5 weeks cumul	26203.	3554.88	{675., 6428.}	90.0894	1.15572	newly 50 weeks cumul 35015. 15107.6 {1313., 83303.} 94.834 0.989194
13	newly 3 weeks spec	5099.	2859.63	{75., 8007.}	112.883	1.16879	newly 3 weeks cumul	17831.	7419.49	{436., 27331.}	96.148	1.02811	newly 5 weeks spec	3072.	1165.95	{7., 4311.}	82.2204	1.27421	newly 5 weeks cumul	24961.	10445.1	{647., 37445.}	94.8581	1.01119	newly 50 weeks cumul 33743. 22512.7 {1013., 83401.} 97.2372 0.91688
14	newly 3 weeks spec	4962.	560.37	{85., 1036.}	92.4794	1.28743	newly 3 weeks cumul	15685.	2144.68	{489., 2414.}	91.3827	1.14894	newly 5 weeks spec	3640.	490.85	{8., 2086.}	87.4981	1.42681	newly 5 weeks cumul	23643.	3130.12	{526., 5879.}	90.6696	1.15922	newly 50 weeks cumul 36706. 15172.2 {996., 84783.} 89.8494 0.99306
15	newly 3 weeks spec	4360.	724.46	{16., 1034.}	93.3807	1.49133	newly 3 weeks cumul	17059.	3422.62	{496., 4286.}	91.852	1.13153	newly 5 weeks spec	2303.	374.83	{0., 438.}	96.5319	2.00099	newly 5 weeks cumul	22470.	4321.99	{589., 5548.}	90.9758	1.1794	newly 52 weeks cumul 26744. 5277.77 {589., 6568.} 91.6742 1.20969
16	newly 3 weeks spec	5728.	1045.01	{17., 684.}	94.7666	1.46799	newly 3 weeks cumul	18259.	3520.39	{109., 6014.}	91.9122	1.25802	newly 5 weeks spec	4036.	477.99	{4., 618.}	91.6504	1.73456	newly 5 weeks cumul	27272.	4750.94	{145., 9895.}	91.8099	1.32212	newly 51 weeks cumul 38769. 6207.99 {165., 15178.} 90.2963 1.34196
17	newly 3 weeks spec	4479.	1126.29	{27., 2215.}	94.3668	1.26091	newly 3 weeks cumul	20396.	6075.1	{204., 8413.}	98.3657	1.22945	newly 5 weeks spec	1516.	380.38	{8., 1082.}	85.5976	1.20856	newly 5 weeks cumul	24628.	7056.79	{290., 12544.}	95.8332	1.20445	newly 57 weeks cumul 26798. 8700.78 {302., 16228.} 95.0838 1.13645
18	newly 3 weeks spec	4189.	1011.79	{0., 2127.}	89.8804	1.83362	newly 3 weeks cumul	17056.	16534.4	{2336., 33373.}	85.0314	0.445772	newly 5 weeks spec	1958.	513.7	{0., 1142.}	93.6415	2.56859	newly 5 weeks cumul	22027.	17727.6	{2992., 43804.}	80.9857	0.483326	newly 55 weeks cumul 25613. 19739.1 {4832., 47008.} 77.4787 0.475479
19	newly 3 weeks spec	3570.	834.94	{0., 1494.}	93.7053	1.88329	newly 3 weeks cumul	17052.	13685.5	{1631., 14408.}	95.6964	0.57172	newly 5 weeks spec	1298.	419.36	{0., 999.}	95.6071	2.58372	newly 5 weeks cumul	20704.	14679.1	{1631., 15888.}	92.84	0.604769	newly 55 weeks cumul 22668. 16114.8 {1631., 23198.} 90.0696 0.595414
20	newly 3 weeks spec	4499.	674.32	{124., 979.}	93.3879	1.23112	newly 3 weeks cumul	19401.	5899.92	{1112., 5813.}	93.1322	0.910293	newly 5 weeks spec	1849.	206.42	{9., 296.}	96.9811	1.6178	newly 5 weeks cumul	24272.	6459.68	{1256., 6681.}	93.2233	0.9568	newly 53 weeks cumul 27031. 6816.75 {1298., 7153.} 92.9618 0.975734
21	newly 3 weeks spec	5237.	1276.99	{214., 1665.}	89.2631	0.991475	newly 3 weeks cumul	23703.	7067.58	{1030., 8580.}	94.0886	0.967448	newly 5 weeks spec	1742.	463.61	{60., 843.}	81.4839	0.909419	newly 5 weeks cumul	28600.	8234.91	{1255., 10088.}	91.2981	0.94222	newly 57 weeks cumul 30632. 10073.6 {1688., 11342.} 89.6532 0.873628
22	newly 3 weeks spec	5006.	908.43	{0., 1860.}	90.9519	2.19734	newly 3 weeks cumul	21806.	10257.	{750., 14383.}	82.7763	0.741477	newly 5 weeks spec	1880.	511.61	{0., 1458.}	91.309	2.54822	newly 5 weeks cumul	26703.	11426.2	{750., 14383.}	82.3144	0.775026	newly 55 weeks cumul 29097. 13439.7 {750., 17802.} 80.2314 0.755782
23	newly 3 weeks spec	1987.	641.49	{15., 442.}	108.421	1.33298	newly 3 weeks cumul	17807.	3201.79	{362., 2333.}	93.6405	1.154	newly 5 weeks spec	308.	254.65	{0., 182.}	144.01	1.58912	newly 5 weeks cumul	18940.	3882.75	{390., 2740.}	95.5918	1.15022	newly 59 weeks cumul 19129. 4235.48 {404., 3052.} 96.4192 1.1399
24	newly 3 weeks spec	4945.	798.41	{42., 800.}	95.9375	1.47727	newly 3 weeks cumul	26504.	6391.91	{516., 10639.}	90.4947	1.06961	newly 5 weeks spec	1389.	218.62	{1., 147.}	99.9338	1.90784	newly 5 weeks cumul	30352.	7007.72	{537., 10666.}	90.4313	1.10051	newly 60 weeks cumul 31658. 7316.06 {554., 10666.} 91.0485 1.11391
25	newly 3 weeks spec	3439.	792.4	{0., 862.}	99.1753	1.61079	newly 3 weeks cumul	21402.	6746.46	{614., 12202.}	89.9793	0.961082	newly 5 weeks spec	698.	211.7	{0., 121.}	110.619	1.75357	newly 5 weeks cumul	23735.	7353.37	{657., 12567.}	90.833	0.984654	newly 60 weeks cumul 24404. 7653.83 {678., 12595.} 91.6106 0.992142
26	newly 3 weeks spec	5656.	1382.09	{0., 1666.}	86.5812	1.58718	newly 3 weeks cumul	23609.	13543.2	{3888., 18911.}	82.184	0.579065	newly 5 weeks spec	2372.	372.	{0., 605.}	92.5911	1.73941	newly 5 weeks cumul	29758.	14632.4	{4151., 20205.}	82.2656	0.63165	newly 54 weeks cumul 33191. 15120.1 {4211., 20277.} 81.9313 0.657091
27	newly 3 weeks spec	4510.	830.24	{0., 1683.}	93.0687	2.02025	newly 3 weeks cumul	21137.	12550.2	{407., 35884.}	86.5537	0.707766	newly 5 weeks spec	1681.	459.07	{0., 1085.}	98.1077	2.81548	newly 5 weeks cumul	25596.	13607.5	{407., 43007.}	85.685	0.738988	newly 55 weeks cumul 27946. 15399.1 {465., 43007.} 84.4708 0.719877
28	newly 3 weeks spec	3624.	1579.69	{0., 3447.}	82.4009	1.35991	newly 3 weeks cumul	14491.	13871.4	{3086., 29765.}	89.9496	0.443284	newly 5 weeks spec	2108.	346.68	{0., 542.}	90.4943	1.62268	newly 5 weeks cumul	19406.	14948.5	{3209., 31605.}	83.517	0.49311	newly 54 weeks cumul 24332. 15292.3 {3232., 31814.} 80.8916 0.551386
29	newly 3 weeks spec	4912.	835.48	{0., 1698.}	94.23	2.06145	newly 3 weeks cumul	20248.	13504.5	{1004., 31666.}	92.0702	0.664795	newly 5 weeks spec	2132.	427.4	{0., 1137.}	94.772	2.90019	newly 5 weeks cumul	25828.	14518.4	{1030., 32271.}	88.8702	0.708787	newly 55 weeks cumul 29513. 16025.5 {1030., 32271.} 86.0121 0.706202
30	newly 3 weeks spec	4348.	1286.11	{22., 707.}	108.508	1.39248	newly 3 weeks cumul	16097.	3794.54	{340., 4850.}	93.2981	1.16867	newly 5 weeks spec	2202.	409.93	{7., 388.}	100.075	1.61206	newly 5 weeks cumul	21394.	5063.26	{428., 6149.}	95.068	1.20524	newly 51 weeks cumul 26261. 5574.44 {480., 7309.} 93.3867 1.21712
31	newly 3 weeks spec	1715.	925.18	{69., 780.}	114.535	1.01034	newly 3 weeks cumul	14771.	7154.03	{798., 11429.}	97.1446	0.855601	newly 5 weeks spec	342.	199.15	{3., 170.}	127.026	1.27549	newly 5 weeks cumul	15873.	7775.7	{849., 11555.}	98.4898	0.864726	newly 60 weeks cumul 16071. 7986.31 {866., 11561.} 99.1003 0.864454
32	newly 3 weeks spec	4733.	1269.28	{225., 2127.}	83.9155	0.909625	newly 3 weeks cumul	23783.	8626.18	{2455., 16947.}	78.4875	0.67126	newly 5 weeks spec	1187.	464.77	{8., 977.}	77.3538	1.00202	newly 5 weeks cumul	27483.	9824.84	{2727., 18122.}	77.5399	0.662924	newly 58 weeks cumul 28771. 10925.3 {2727., 18265.} 76.4286 0.634854
33	newly 3 weeks spec	3999.	1183.6	{140., 1996.}	77.9885	0.874426	newly 3 weeks cumul	20824.	8201.49	{2539., 9751.}	79.9547	0.647451	newly 5 weeks spec	1142.	427.43	{3., 989.}	67.4299	1.00115	newly 5 weeks cumul	24133.	9311.92	{2824., 10921.}	78.5077	0.640294	newly 58 weeks cumul 25686. 10230.9 {2878., 13605.} 75.9577 0.614404
34	newly 3 weeks spec	4878.	1108.11	{185., 1742.}	86.8141	0.97123	newly 3 weeks cumul	19185.	3757.93	{768., 6523.}	88.0906	1.00148	newly 5 weeks spec	2525.	948.41	{63., 2282.}	75.2566	0.861041	newly 5 weeks cumul	25181.	5716.61	{1054., 9919.}	85.4731	0.932922	newly 50 weeks cumul 30277. 18688.1 {1872., 83999.} 87.7464 0.747382
35	newly 3 weeks spec	3516.	700.66	{7., 411.}	104.254	1.61424	newly 3 weeks cumul	21757.	2544.95	{424., 2042.}	93.1574	1.31043	newly 5 weeks spec	674.	267.68	{0., 168.}	116.923	1.92398	newly 5 weeks cumul	24081.	3288.23	{425., 2471.}	95.0542	1.32101	newly 59 weeks cumul 24720. 3630.64 {425., 2776.} 95.6183 1.314
36	newly 3 weeks spec	6059.	1271.07	{164., 2309.}	88.8528	1.07032	newly 3 weeks cumul	27874.	7197.2	{1020., 9356.}	93.3472	1.05217	newly 5 weeks spec	2044.	470.58	{36., 850.}	83.3112	0.971758	newly 5 weeks cumul	33550.	8377.88	{1281., 13443.}	90.8392	1.0245	newly 57 weeks cumul 35952. 10137.9 {1494., 15224.} 89.3587 0.954906
37	newly 3 weeks spec	2491.	647.07	{9., 390.}	106.113	1.41223	newly 3 weeks cumul	16769.	3085.11	{485., 2096.}	93.6122	1.13955	newly 5 weeks spec	544.	268.18	{0., 165.}	123.787	1.68716	newly 5 weeks cumul	18580.	3788.57	{487., 2439.}	95.8736	1.14194	newly 59 weeks cumul 19084. 4181.57 {487., 2829.} 96.9786 1.13773
38	newly 3 weeks spec	4614.	1432.1	{437., 2111.}	84.7295	0.792179	newly 3 weeks cumul	23415.	7826.55	{1791., 8653.}	90.3211	0.815157	newly 5 weeks spec	1497.	521.85	{117., 1182.}	66.5097	0.661186	newly 5 weeks cumul	27574.	9161.5	{2499., 11254.}	87.3183	0.77889	newly 57 weeks cumul 29436. 10752.6 {3165., 15115.} 82.3516 0.703658
39	newly 3 weeks spec	3711.	2182.87	{0., 3224.}	91.9863	1.22012	newly 3 weeks cumul	14691.	13923.8	{3370., 32405.}	84.4719	0.407401	newly 5 weeks spec	1875.	428.15	{0., 580.}	87.4064	1.40958	newly 5 weeks cumul	19116.	15342.8	{3535., 34400.}	79.6923	0.443456	newly 54 weeks cumul 22836. 15727.5 {3535., 34714.} 77.8288 0.485466
40	newly 3 weeks spec	4019.	1212.35	{53., 1415.}	98.3645	1.2016	newly 3 weeks cumul	16800.	6239.71	{225., 7017.}	101.551	1.13609	newly 5 weeks spec	1644.	430.41	{17., 622.}	90.4617	1.20642	newly 5 weeks cumul	20945.	7343.4	{300., 8769.}	99.5884	1.12835	newly 57 weeks cumul 23590. 8635.05 {421., 10615.} 98.2156 1.0863
41	newly 3 weeks spec	3584.	636.14	{7., 425.}	101.451	1.52957	newly 3 weeks cumul	19699.	3418.02	{389., 4735.}	91.815	1.16844	newly 5 weeks spec	987.	264.5	{0., 160.}	109.824	1.93827	newly 5 weeks cumul	22587.	4100.94	{391., 4770.}	93.5786	1.19326	newly 59 weeks cumul 23519. 4482.41 {391., 5208.} 94.4594 1.19432
42	newly 3 weeks spec	4951.	803.49	{8., 806.}	98.8889	1.67403	newly 3 weeks cumul	19337.	3835.59	{157., 3584.}	97.9746	1.28503	newly 5 weeks spec	2604.	427.52	{0., 381.}	97.1905	2.15332	newly 5 weeks cumul	25605.	4845.65	{157., 4569.}	96.9568	1.32799	newly 52 weeks cumul 30663. 6239.25 {157., 5079.} 95.2978 1.342
43	newly 3 weeks spec	3668.	1198.5	{57., 619.}	107.183	1.2034	newly 3 weeks cumul	13660.	3570.41	{478., 3216.}	90.3985	1.00172	newly 5 weeks spec	2047.	506.56	{9., 305.}	102.474	1.45098	newly 5 weeks cumul	18398.	5035.49	{480., 3811.}	93.7094	1.04099	newly 51 weeks cumul 23176. 5772.68 {480., 4648.} 91.0195 1.03599
44	newly 3 weeks spec	5502.	1180.27	{186., 2202.}	81.966	0.947347	newly 3 weeks cumul	26971.	8626.	{2664., 19686.}	74.0716	0.666475	newly 5 weeks spec	1807.	459.95	{9., 972.}	81.8545	1.19366	newly 5 weeks cumul	31936.	9798.39	{2927., 22548.}	74.0405	0.669653	newly 58 weeks cumul 33949. 10949.6 {2951., 22813.} 73.6455 0.64906
45	newly 3 weeks spec	5745.	2363.7	{75., 8028.}	97.0938	1.09686	newly 3 weeks cumul	23088.	16131.9	{817., 59148.}	102.758	0.77893	newly 5 weeks spec	2549.	551.63	{3., 858.}	95.3084	1.55736	newly 5 weeks cumul	29573.	17763.7	{1081., 67178.}	99.3141	0.829229	newly 56 weeks cumul 33764. 18906.5 {1241., 68009.} 97.7139 0.855088
46	newly 3 weeks spec	5945.	822.81	{0., 1058.}	94.813	2.08512	newly 3 weeks cumul	25387.	12092.1	{863., 24169.}	76.5907	0.702039	newly 5 weeks spec	2372.	442.84	{0., 548.}	98.2437	2.76701	newly 5 weeks cumul	31718.	13108.	{1216., 24176.}	78.8155	0.741678	newly 55 weeks cumul 35057. 14896.9 {1751., 24176.} 78.9251 0.703911
47	newly 3 weeks spec	4772.	2021.04	{36., 10140.}	92.9497	1.1388	newly 3 weeks cumul	17003.	5789.93	{194., 18717.}	80.3642	1.00885	newly 5 weeks spec	2927.	1739.06	{13., 12336.}	114.886	1.30828	newly 5 weeks cumul	23808.	9550.69	{268., 44487.}	87.3819	1.0585	newly 51 weeks cumul 31769. 13461.3 {730., 67217.} 86.5312 1.00474
48	newly 3 weeks spec	5735.	2520.84	{60., 10742.}	100.302	1.11835	newly 3 weeks cumul	21931.	14856.1	{474., 50475.}	100.393	0.788222	newly 5 weeks spec	2847.	633.21	{6., 1441.}	94.4542	1.59112	newly 5 weeks cumul	29108.	16696.2	{633., 56132.}	97.4577	0.848064	newly 56 weeks cumul 34186. 17937.5 {739., 68721.} 95.8466 0.878946
49	newly 3 weeks spec	3521.	765.01	{11., 778.}	97.9651	1.41224	newly 3 weeks cumul	23225.	6418.21	{138., 11521.}	90.7237	1.1273	newly 5 weeks spec	685.	203.75	{0., 147.}	108.731	1.75221	newly 5 weeks cumul	25469.	7001.05	{140., 11652.}	91.3936	1.1451	newly 60 weeks cumul 25996. 7277.73 {140., 11656.} 92.1098 1.15002
50	newly 3 weeks spec	2974.	646.6	{9., 372.}	103.13	1.42464	newly 3 weeks cumul	18646.	3289.83	{395., 4120.}	91.8277	1.11887	newly 5 weeks spec	685.	269.53	{0., 150.}	117.485	1.72129	newly 5 weeks cumul	20804.	3994.14	{403., 5157.}	93.9906	1.13311	newly 59 weeks cumul 21381. 4394.46 {403., 5939.} 95.0166 1.12877
